# Neurocognitive mechanisms of reactions to second- and third-party justice violations

**DOI:** 10.1038/s41598-019-45725-8

**Published:** 2019-06-25

**Authors:** Claudia Civai, Inge Huijsmans, Alan G. Sanfey

**Affiliations:** 1 Division of Psychology, School of Applied Science, London South Bank University, London, UK; 2 Donders Institute for Brain, Cognition and Behavior, Radboud University, Nijmegen, The Netherlands; 3 Behavioural Science Institute, Radboud University, Nijmegen, The Netherlands

**Keywords:** Social behaviour, Decision

## Abstract

The aim of the current study was threefold: (i) understand people’s willingness to engage in either punishment of the perpetrator or compensation of the victim in order to counteract injustice; (ii) look into the differences between victims of and witnesses to injustice; (iii) investigate the different role played by social preference and affective experience in determining these choices. The sample tested here showed an equal preference for punishment and compensation; neuroimaging findings suggested that compensation, as opposed to punishment, was related to Theory of Mind. Partially supporting previous literature, choosing how to react to an injustice as victims, rather than witnesses, triggered a stronger affective response (striatal and prefrontal activation). Moreover, results supported the idea that deciding whether or not to react to an injustice and then how severely to react are two distinct decisional stages underpinned by different neurocognitive mechanisms, i.e., sensitivity to unfairness (anterior insula) and negative affectivity (amygdala). These findings provide a fine-grained description of the psychological mechanisms underlying important aspects of social norm compliance.

## Introduction

Norms such as fairness and cooperation are fundamental principles of society, and in recent years there has been a dramatic increase in investigations of the psychological underpinnings of these social norms. Many experimental investigations have demonstrated people’s willingness to spend personal resources, in sometimes significant amounts, to promote norm compliance. For example, people often reject an unfair division of resources by punishing the proposer of an unfair offer, even when this act is personally financially costly^1–3^. Importantly, these rejections happen even when people are not the target of injustice themselves, but are instead deciding on behalf of a third-party^4,5^. This behaviour has typically been termed ‘costly altruistic punishment’, denoting the idea that people are often willing to override their own immediate self-interest in order to promote compliance with social norms, both when directly (i.e. Second Party (SP) punishment^2,3^) and non-directly (i.e. Third Party (TP) punishment^5^) affected by norm violations. Using one’s own resources to punish offenders is one strategy to enforce compliance - for example, most of us pay taxes which in turn finances the prison system. However, an alternative approach to emphasizing cooperative norms is that of compensating the victims of injustice, where fairness can be re-established by assisting the victim, offering aid to increase their depleted resources even though this aid comes at a personal cost^6–11^.

Although both of these approaches are reactions to a social transgression, these strategies differ substantially in terms of output: punishment discourages social norm violation by harming offenders and leaving them worse off, whereas compensation focuses on victims, signalling altruism and prosociality^12^. Despite these differences, and the obvious societal impact of these two methods of norm enforcement, relatively few comparative studies have been conducted exploring how these putative mechanisms of norm compliance may either overlap or differ in terms of their neural processes.

Therefore, the first goal of the current study was to investigate the neural processing of punishment and compensation. Recent research has begun to shed some light on the neural mechanisms underlying these processes, though several questions are still outstanding. In one recent study, Stallen and colleagues developed a novel task, termed the Justice Game, and found that when confronted with an obvious norm violation and then given the opportunity to take action either towards perpetrator or the victim, people typically preferred to punish the perpetrator than to compensate the victim^11^. This behavioural preference correlated with increased activation of the ventral striatum, one of the main components of the brain reward system^13^, consistent with previous research highlighting the rewarding nature of altruistic punishment^3,14^. However, in a separate study, Hu and colleagues found no behavioural difference between punishment and compensation rates, and also found common activation of the bilateral striatum across conditions, suggesting that reacting to injustice may always be perceived as ‘rewarding’^8^; on the other hand, Theory of Mind (ToM) areas such as temporo-parietal junction (TPJ)^15^ were associated more with compensation than punishment. This, and other findings^9,10^, suggests that compensation may be driven more by prosocial and other-regarding motivations.

However, though the tasks employed across these studies were superficially similar, there are some important differences to take into account: Stallen and colleagues offered participants certain trials in which they could punish and others in which they could compensate, allowing for separate assessment of the propensity to carry out each action, but which makes direct comparison between the two more difficult^11^; conversely, Hu and colleagues required their participants to always choose between either punishment and compensation^8^. Here, we developed a modified version of the aforementioned Justice Game, in which participants sometimes directly chose between punishment and compensation, and other times made a decision with only one of the two options available. This novel design enabled us to determine the preference for punishment or compensation when directly comparing the two (as in)^8^, as well as the general willingness to react to injustice when only one action is available (as in)^11^. This allowed for the categorising of participants based on their subjective preference, and the investigation of the mechanisms involved when their preferred option is, or is not, available. Neural data can then help to determine the extent to which motivations of reward (striatum) and prosociality (ToM areas) are involved in the choice. We hypothesised that reward areas were associated with a preference for punishment, whereas ToM areas were associated with a preference for compensation.

Costly altruistic punishment has been studied using laboratory-based Game Theory paradigms and hypothetical crime scenarios^16^. Economic games have been used to investigate both SP punishment^1,3,17^, i.e., when the victim of a social norm violation is given the chance to punish the transgressor, and TP punishment^4,5^, i.e., when the witness of a violation is given the chance to punish the transgressor, whereas crime scenarios have been mostly employed to investigate TP punishment^18–21^. Recent meta-analyses of the neural basis of altruistic punishment suggested that TP punishment is an extension of SP punishment, and that the two share the same cognitive processes and neural underpinnings: sensitivity to norm violation and norm compliance (anterior insula (AIns) and dorsolateral prefrontal cortex (DLPFC)), negative affect (amygdala) and integration between self-referential (medial prefrontal cortex (MPFC) and posterior cingulate cortex (PCC)) and other-regarding (TPJ) processes^16,18^. Nevertheless, previous neuroimaging research directly comparing SP and TP punishment has shown both similarities and differences between the two: in terms of neural activity, stronger sensitivity to fairness norm violations and norm compliance, observed as increased AIns and DLPFC activation, can explain the rejection of unfair actions of another, as well as punishment, both in SP and in TP^16,17,22–25^. However, people tend to exhibit different behaviours, with higher punishment rates observed when they themselves are victims (SP), as compared to when they witness (TP)^5,11^ an injustice; moreover, ventral striatum has been found to be more active for SP as compared to TP, suggesting that the rewarding sensation associated with punishment is heightened with personal involvement^11,14^. The second goal of this study was, therefore, to compare Second Party (SP) and Third Party (TP) punishment. Exploring how neural processing may be similar (and different) across these two conditions can help in disentangling mechanisms associated with general punishment processes and ones that are tied to personal involvement. We aimed to collect further evidence to investigate whether two different mechanisms, i.e., sensitivity to unfairness and reward, contribute to explaining SP and TP punishment differences: our task allowed for direct comparisons between SP and TP conditions when evaluating the extent of the injustice (when participants see the actions of others) as well as when making a decision (when they decide whether or not to act upon injustice). Based on previous studies, we expected that participants would punish more in SP, and that reward areas would be more active when choosing in the SP compared to the TP condition^11,14^. On the other hand, we hypothesised no difference between SP and TP in the activation of areas associated with sensitivity to unfairness and norm compliance (AIns, DLPFC) when the division is evaluated^22,23^.

Finally, the third objective of this study was to distinguish between two potentially different motives underpinning why we react to unfairness and injustice: social preference and affective experience. As proposed by^11^, the decision to react to injustice is underpinned by social motivations, such as inequality aversion^17,26^, whereas the severity of the reaction, i.e., how much people are willing to punish, might rather be linked to an affective experience, such as anger and frustration associated with unfairness^27^. Adapting^11^, the novel paradigm we used here allowed for the assessment of two psychologically distinct decision stages, one where participants indicated whether or not they want to act upon the injustice (response selection), and a second when they decided the severity of the punishment/compensation (how much they want to spend). We hypothesized that, if different motives act in distinct stages of the decision process, unfairness-related areas such as AIns^17,22,23^ would correlate with the decision to react to injustice, whereas areas associated with negative affective experience, such as amygdala^28^, would correlate with the amount of money spent to punish or compensate.

## Results

### Behavioral results

**Figure 1:**
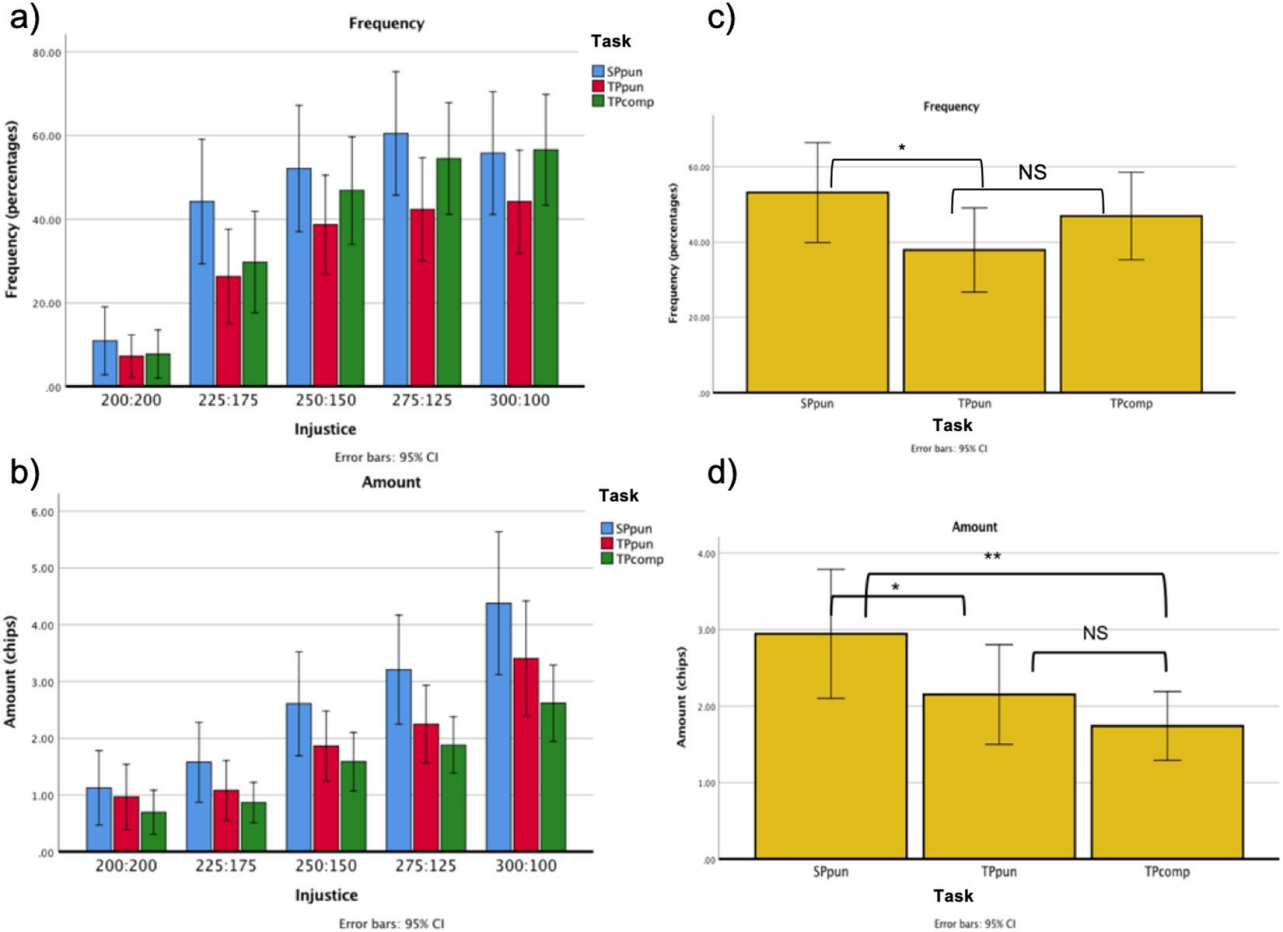
Behavioral results: (**a**) frequency of reaction and (**b**) amount spent for Task (Second Party (SP) punishment, Third Party (TP) punishment, TP compensation) and Injustice level represented in terms of amount of chips owned by both players A and B after player A’s decision (0 chips taken, or 200:200 = fair division; 100 chips taken, or 300:100 = extremely unfair division); (**c**,**d**) frequencies and amounts for each Task (SPpun; TPpun; TPcomp) for unfair trials (injustice levels collapsed across unfair divisions; NS = non-significant difference, *p < 0.05, **p < 0.01).

The target (SP or TP), the type of reaction to injustice made available (punish in SP; punish, compensate or both in TP), and the level of the injustice (0, 25, 50, 75, 100 coins could be taken from either the participant or the third-party) were manipulated. We measured the frequency of each reaction and the amount spent. No participant was excluded from the behavioural analysis. Four participants never reacted to injustice and always chose to Leave; one participant never reacted in the TP condition, and two never reacted in the SP condition; four participants never punished, neither in SP nor in TP, but did compensate; two participants never punished in TP, but did compensate; three participants never compensated, but did punish in TP. The remaining 22 participants chose to compensate and punish in SP and TP at least once. Analyses were performed using SPSS 25^29^. A repeated-measure Multivariate Analysis of Variance (MANOVA) was run considering two factors: Task and Injustice. Task had 3 levels: Second Party punishment (SPpun), Third Party punishment (TPpun), Third Party compensation (TPcomp). The analysis was limited to TP trials where only 2 options were available, i.e., the choice between punishment and no reaction (Leave or Take (LT) trials), or between compensation and no reaction (Leave or Give (LG) trials). Injustice had 5 levels: 0, 25, 50, 75, 100 out of 200. These were the amount of chips that player A could steal from either the participant (in SP condition) or player B (in TP condition). Frequency of reaction to injustice and amount spent to react were the dependent variables. Order of presentation was considered a between-participants factor. A significant main effect of Injustice was found both for frequency and for amount (frequency: F(4, 144) = 63.18, p < 0.001, partial η^2^ = 0.63; amount: F(4, 144) = 40.08, p < 0.001, partial η^2^ = 0.53), indicating that participants are more willing to act as a function of the level of injustice, and spend more as the injustice becomes greater. A significant main effect of Task was found for amount (F(2, 72) = 8.44, p < 0.001, partial η^2^ = 0.19), but not for frequency (Fig. 1a,b). Because of our interest in comparing SP and TP punishment, and TP punishment and compensation, planned comparisons were performed on both amount and frequency; the results of these comparisons, Bonferroni-corrected, indicated a significant difference between both SPpun and TPpun amounts (t(37) = 2.68, p < 0.05, Cohen’s d = 0.44), and SPpun and TPpun frequencies (t(37) = 2.91, p < 0.05 Cohen’s d = 0.47), across injustice levels (Fig. 1c,d). A post hoc comparison for the amount also revealed a significant difference between SPpun and TPcomp amounts (t(37) = 3.6, p < 0.01, Cohen’s d = 0.58). No difference between TPpun and TPcomp was found, neither for frequency nor for amount. Two t-tests were performed to analyse the difference between TP punishment and compensation frequencies in relation to the number of options (2-option or 3-option conditions): punishment in LT trials (i.e., only punishment option) was compared with compensation in LG trials (i.e., only compensation option), and punishment and compensation were compared in Leave-Take-Give (LTG) trials (i.e., both options available). No significant difference was found, suggesting an equal preference for the two strategies in our sample. No effect of order of presentation was found. Given the deceptive nature of the task, participants were asked at the end if they had any doubt about the plausibility of the final outcome and, whilst some were sceptical, most of them did not report any doubt.

### fMRI results

In addition to whole-brain analysis, given the clear brain location hypotheses based on previously discussed literature results, small volume correction (svc) was applied to the contrasts of interest. The following anatomical masks, built with Neuromorphometrics Atlas (MRI scans originated by the OASIS project, and labelled data provided by Neuromorphometrics, Inc), were used to determine the small volumes of interest: (1) AIns and Middle Frontal Gyrus (DLPFC) (unfairness sensitivity and norm compliance) for contrasts determining unfairness-related activation, i.e., main effect of injustice; (2) nucleus accumbens, caudate, putament (i.e., striatum, reward-related area) and angular gyrus and supramarginal gyrus (i.e., TPJ, ToM area) for contrasts looking at differences between punishment and compensation, for both the *Option* and the *Choice* model (see Methods section); (3) AIns, Middle Frontal Gyrus (DLPFC) and striatum, for contrasts comparing SP and TP, both when evaluating unfairness (*Division* screen) and when responding to it (*Response* screen), for both the *Option* and the *Choice* model; (4) AIns and amygdala (affective response) for contrasts looking at the differences between motives underlying reaction to injustice, i.e., *Response* selection and *Amount* selection. All results are reported using a common cluster-corrected threshold at p < 0.05 FWE on the basis of a whole-brain voxel-wise threshold of p < 0.001, unless otherwise specified in the tables (svc, cluster-corrected threshold at p < 0.05 FWE).

The reason why we have selected the current areas is that the aim was to focus on specific cognitive processes hypothesised to be involved in the behaviours of interest: reward and prosociality in punishment and compensation, sensitivity to injustice and reward in second-party versus third-party condition, and sensitivity to injustice and negative affect in response selection versus severity of the response. We have selected the brain areas associated with these processes based on previous studies^8,11^ using the same or a very similar paradigm. Nevertheless, the results of whole-brain analyses are reported, and the involvement of other relevant areas reported in recent meta-analyses^16,18^, such as MPFC and PCC, is discussed.

#### Comparison between punishment and compensation in response selection

No significant cluster was found when contrasting activations associated with only-punishment and only-compensation option (*RespTP_LT-RespTP_LG* contrast, or its reverse, *Option* model). When considering participants’ choices (*Choice* model), no significant cluster was found for punishing vs compensating choices (*RespTP*_*pun*_*-RespTP*_*comp*_); the reverse contrast, i.e., compensating vs punishing choices (*RespTP*_*comp*_*- RespTP*_*pun*_), returned activation in a small cluster of left TPJ, which, however, did not survive svc.

**Figure 2:**
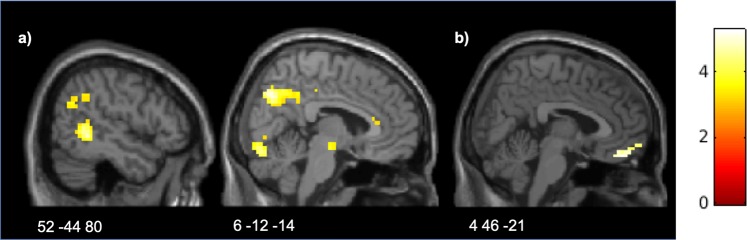
Areas associated with preference for punishment and compensation: (**a**) Activation of TPJ, MidTG and precuneus when Compensators, as opposed to Punishers, selected a response; (**b**) Activation of VMPFC associated with response selection when the preferred option is the only one available. Activation maps are superimposed on an MNI template.

The full factorial analysis showed significant clusters in the precuneus, right TPJ and right middle temporal gyrus (MidTG) for Compensators compared to Punishers, supporting the idea that ToM areas are more involved in compensation (Fig. 2a, Table 1a). No significant cluster was found for the reverse main effect of Preference (Punishers vs Compensators). When the preferred option was available (Compensators could compensate (LG trials) and Punishers could punish (LT trials)), one cluster was active in ventromedial prefrontal cortex (VMPFC) (Fig. 2b, Table 1b), which survived svc (anatomical medial prefrontal gyrus used as small volume). Although VMPFC was not in our original list of regions of interest, this finding is worth of mention given the widely reported involvement of this area in subjective value-based choices and affect computations^30^. For the reverse contrast, i.e., when Compensators could only punish and Punishers could only compensate, no cluster survived neither whole-brain correction nor svc.

**Table 1:** Significant clusters correlating with Punishment vs Compensation (peak MNI coordinates are reported; svc: small volume correction, based on a-priori defined anatomical areas).

Brain region	Lateral	x	y	z	K (cluster size)	Z	p_fwe-corr_
(***a***) ***Compensators-Punishers*** (***Option*** **model**, **full factorial**)							
Precuneus	R	4	−70	35	154	4.90	<0.001
MidTG	R	50	−46	−4	135	4.50	0.002
Angular gyrus (TPJ)	R	43	−60	24	30	3.72	0.039 (svc)
(***b***) **Preferred choice exclusively available** ***-*** **(*Compensators_LG* + *Punishers_LT*)*-rest*** **(*Option*** **model, full factorial)**							
VMPFC	R	4	46	−21	20	3.89	0.024 (svc)

#### Comparison between SP and TP

Injustice perception. The contrast between unfair vs fair divisions (*Div*_*Unfair*_*-Div*_*Fair*_) was associated with significant clusters in the DLPFC and AIns (Table 2a). A significant whole-brain activation of the fusiform gyrus and the superior parietal lobule was found when comparing SP to TP. No suprathreshold activation was found in the selected ROIs, and no difference was found between SP and TP when processing unfair divisions (*DivSP*_(*Unfair-Fair*)_*-DivTP*_(*Unfair-Fair*)_).

**Table 2:** Significant clusters correlating with second-party (SP) and third-party (TP) in Division and Response screen (peak MNI coordinates are reported; svc: small volume correction, based on a-priori defined areas).

Brain region	Lateral	x	y	z	K (cluster size)	Z	p_fwe-corr_
(***a***) **Main effect of Injustice** ***- Div***_***Unfair***_***-Div***_***Fair***_ (***Option*** **model**)							
Superior Parietal Lobule	R	29	−63	38	82	Inf	0.013
Superior Parietal Lobule	L	−30	60	46	92	Inf	0.009
Thalamus	R	22	−28	0	151	4.71	0.001
Pallidum	L	−10	7	4	162	4.66	0.001
DLPFC – Middle Frontal Gyrus	L	−41	7	32	27	4.84	0.041 (svc)
AIns	L	−34	18	4	23	5.85	0.05 (svc)
AIns	R	32	21	7	24	5.47	(svc)
(***b***) **Main effect of Target** ***– Div***_***SP***_***-Div***_***TP***_ (***Option*** **model**)							
Fusiform Gyrus	L	−38	−52	−18	269	4.96	<0.001
Fusiform Gyrus	R	40	−56	−18	120	4.31	<0.001
Superior Parietal Lobule	R	29	−66	32	79	4.15	0.008
Inferior Occipital Gyrus	R	32	−91	0	72	3.97	0.012
(***c***) **Deciding in SP vs TP** ***- RespSP-RespTP_LT*** (***Option*** **model**)							
MPFC/ACC	L	−6	38	7	198	4.94	<0.001
Middle Occipital Gyrus	L	−48	−77	7	113	6.14	<0.001
Precuneus	L	6	−56	14	78	4.63	0.013
Posterior cingulate gyrus	L	−2	32	46	150	4.83	0.001
Planum Temporale	R	54	−32	21	758	5.54	<0.001
Superior Temporal Gyrus	L	−62	−10	4	719	5.47	<0.001
Striatum -Putamen	R	29	−14	7	62	4.49	0.006 (svc)
Striatum -Putamen	L	−24	4	−10	45	4.41	0.016 (svc)
(***d***) **Deciding in TP vs SP** ***– RespTP_LT-RespSP*** (***Option*** **model**)							
Calcarine Cortex	L	−13	−91	−7	723	6.90	<0.001
Superior Parietal Lobule	R	29	−63	38	80	4.38	<0.001
(***e***) **Deciding to punish in SP vs TP** ***– RespSP***_***pun***_***-RespTP***_***pun***_ (***Choice*** **model**)							
ACC/White matter	R	22	38	10	89	4.60	0.005

**Figure 3:**
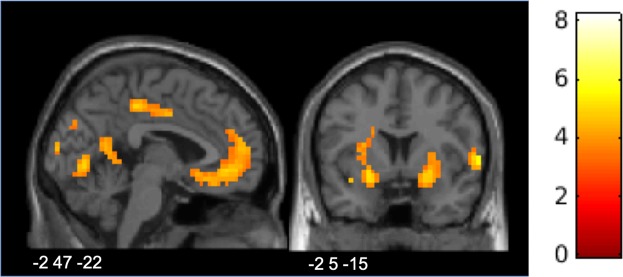
Areas associated with responding in Second Party (SP) as opposed to Third Party (TP): activation of MPFC and striatum. Activation map is superimposed on an MNI template.

Response selection. When comparing the response time-window in SP and TP (*Option* model, contrast *RespSP-RespTP_LT*), clusters in the PCC, the dorsolateral striatum, and the MPFC/anterior cingulate cortex (ACC) were more active in SP compared to TP (Fig. 3 and Table 2b). Significant clusters in the occipital and parietal areas were found both for *RespSP-RespTP_LT* and for the reverse contrast (Table 2c). These activations are likely due to the difference in the visual appearance of the stimuli (words) in SP and TP. A significant cluster in a region encompassing the ACC and the right medial and dorsal PFC, as well as white matter, was found when participants decided to punish in SP compared to TP (*Choice* model, contrast *RespSP*_*pun*_*-RespTP*_*pun*_; Table 2d). No significant activation was found for the reverse contrast, i.e. when participants decided to punish in TP compared to SP.

#### Differences between Response Selection and Amount

**Figure 4:**
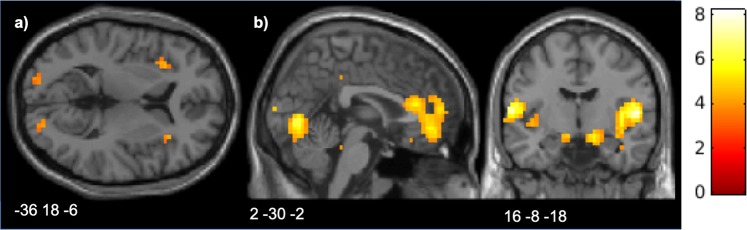
Areas associated with the willingness to react to injustice and the severity of reaction: (**a**) AIns associated with willingness to react; (**b**) ACC/VMPFC and amygdala associated with severity of reaction. Activation maps are superimposed on an MNI template.

A marginally significant activation in the left AIns was associated with the reaction to injustice (*Response* selection) as opposed to the severity of reaction (*Amount* selection) (Fig. 4a, Table 3a). Significant activation in the occipital cortex, most likely associated with the difference in the visual stimulation between the two separate time windows, was also found. The reverse contrast, indicating areas that increase their activation with the severity of the reaction and are more involved in this process compared to response selection, showed significant activations of the MPFC, bilateral central operculum/insula, and the amygdala (Fig. 4b, Table 3b).

**Table 3:** Significant clusters correlating with the difference between Response Selection (willingness to react to injustice) and Amount (severity of reaction) (peak MNI coordinates are reported; svc: small volume correction, based on a-priori defined anatomical areas).

Brain region	Lateral	x	y	z	K (cluster size)	Z	p_fwe-corr_
(**a**) **Response vs Amount** ***-*** **[*Resp_TP***(***pun* + *comp***)** + *Resp_SP*]* − *[*AmTP***(***pun* + *comp***)_***param* + **_***AmSP***_***param***_**]** (***Choice*** **model**)							
Calcarine Cortex	R	15	−88	−4	113	5.44	0.003
Occipital Pole	L	−16	−94	−4	204	3.15	<0.001
AIns	L	−30	21	7	5	3.77	0.06 (svc)
(**b**) **Amount vs Response** ***-*** **[*AmTP*** (***pun* + *comp***)_***param***_** + *AmSP***_***param***_**]* − *[*Resp_TP***(***pun* + *comp***)** + *Resp_SP*]** (***Choice*** **model**)							
Lingual Gyrus	R	1	−74	0	256	4.47	<0.001
Inferior Occipital Gyrus	R	46	−70	7	91	4.22	<0.001
MPFC/ACC	L	−10	46	−10	541	4.51	<0.001
Operculum/PIns	L	−58	−7	7	190	4.86	<0.001
Operculum/PIns	R	54	−4	7	635	5.09	<0.001
Amygdala	R	15	−4	−18	11	4.12	0.036 (svc)

## Discussion

People typically have strong responses when they perceive injustice, and can react in different ways, such as by punishing offenders or compensating victims. These responses have different social consequences, i.e., a decrease in the offenders’ resources or an increase in the victims’ ones, and therefore investigating these decision processes in detail is crucial to better understand the psychosocial elements of justice and equality. In the current study, we addressed three issues: the preference to either punish a perpetrator or compensate a victim; the comparison between a willingness to punish when one is the victim as compared to when one is an unaffected witness; and the process differences between the initial reaction to injustice and the severity of that reaction.

Our participants demonstrated an equal preference for both punishment and compensation. Previous literature is inconclusive on whether there is a dominant strategy to enforce norm compliance. Some evidence points towards a preference for punishment over compensation^11^, whereas other studies have found the opposite pattern^7^. Further, some research shows no overall preference^6,8^, but suggests that individual differences in empathy and perspective taking may explain why some people prefer to compensate. Other personal traits may explain preference for punishment, but further research is needed in this direction. It has been shown that compensation is a more effective act than punishment for reputation enhancement, and thus may be the preferred choice in situations where there is no anonymity in the social exchange^7,12^, that is, where reputation is meaningful. In laboratory studies such as this one, reputation is likely less salient given the anonymous interactions. It is worth noting that this does not diminish findings from lab-based social decision-making studies: on the contrary, a more socially sterile environment may help elicit behaviours and motivations that are otherwise hidden, potentially mimicking the conditions created by internet and social media where people observe and engage in social interactions from an often solitary and isolated space. We acknowledge that some scepticism reported by few participants over the plausibility of the outcomes may have influenced the results; nevertheless, results of experiments using deception are usually in line with experiments where deception is not used^31^.

Based on previous literature, it was hypothesised that punishment would involve the reward system^3,11,14^, whereas compensation would involve perspective taking and ToM^8–10^. The current findings only partially support these hypotheses. We found no evidence that the reward system was specifically associated with punishment as opposed to compensation, which could be explained by the fact that participants did not show a preference for punishing over compensating behaviour in the third-party condition. On the other hand, TPJ and MidTG (ToM areas) were associated with an individual preference for compensation. A recent meta-analysis^15^ found TPJ as the only area involved in all aspects of ToM (from reading emotions from facial expressions to inferring others’ beliefs), whereas MidTG was specifically involved in goal directed tasks, i.e., tasks in which participants need to identify the specific goal to follow an action. Therefore, the involvement of MidTG in compensation may suggest that, in this type of scenario, compensating victims might be regarded as the most suitable action to perform. Importantly, these results suggest that ToM regions are not necessarily associated with the specific choice to compensate versus punish, but rather with the decisional stage in Compensators (individuals who, overall, prefer compensation); this suggests that Compensators engaged ToM abilities more than Punishers when choosing how to react to an injustice, irrespective of the specific choice made. VMPFC activation was correlated with the availability of the preferred option (when Compensators could compensate, and when Punishers could punish): it has been shown that VMPFC activity increases when comparing subjectively valuable options^13,30,32^; hence, this suggests that punishment is not necessarily more rewarding than compensation, or vice-versa, as the two do not have a distinguishable absolute value, but rather a subjective value that depends on individual preferences.

Based on previous literature on second- and third-party punishment, we expected people to punish more, and more harshly, in SP compared to TP condition^5,11,14^. We confirmed this hypothesis, in that we found that participants were willing to punish more and spend more to punish when they were directly damaged by injustice (SP) compared to when they witnessed injustice (TP). Nevertheless, neuroimaging findings support the idea that sensitivity to injustice does not differ between SP and TP^22,23^, since DLPFC and AIns, both of which are associated with unfairness sensitivity^17,18,33,34^, were more active for unfair compared to fair divisions in both SP and TP, with no difference found between the two conditions. The differences between the two conditions lay in activation of the dorsolateral striatum, the PCC and the MPFC/ACC. The striatum was more active when selecting a response in SP compared to TP; however, this activation did not specifically relate to punishing choices. Arguably, the greater involvement of this area in second-party scenarios is associated with decisions made in personally salient social situations^35^, which trigger affective responses irrespective of the value of the specific decision. According to the accounts put forward by [16, 18], the PCC and MPFC, in particular the medial portion, are part of the default mode network modulated by self-referential processes in altruistic punishment. These findings suggest that these processes are more involved in second- versus third-party punishment, possibly also reflecting the increased personal saliency of the SP condition. On the other hand, the ACC and dorsomedial portion of the prefrontal cortex were more active when punishing in SP compared to TP, supporting the idea that this area signals the affective reaction to unfairness and injustice in the presence of personal involvement^36^. Nevertheless, this interpretation remains speculative, given that the activation encompassed a large portion of white matter.

The current design breaks the decision down into multiple steps. One limitation of this is that, in the SP condition, participants could potentially make their decision very early, even at the Division screen stage, since the choice of options was fixed (Take or Leave). We reasoned that if participants did adopt this strategy in SP, decision activity would have increased in the Division screen as compared to the TP condition. Moreover, it would have decreased decision activity in the Response selection screen compared to TP, since participants in SP would have already made their choice by this time. A significant cluster in the superior parietal lobule was found when comparing SP and TP during the Division screen; according to [16, 18], this suggests that, in SP, participants were starting to decide on a punishment as soon as the division was presented. However, no difference between SP and TP was found when considering only the unfair divisions, hence making this interpretation less plausible. On the other hand, neural activity in the Response screen was larger for SP compared to TP in areas which have been associated with self-referential processes in decision-making^16,18,36^. Therefore, we assume that timing of the decisions was not completely shifted forward in SP.

The final goal of this study was to compare two different aspects of the decision process in response to injustice: deciding whether or not to react and, if so, how harshly. The computational model developed by Stallen and colleagues allowed for distinguishing the punishment choice itself from the actual severity of punishment; when applied to neuroimaging data, it was found that AIns correlated with the willingness to punish, whereas amygdala correlated with the severity of punishment. Our results replicate these findings in a different setting, supporting and extending these conclusions. Comparing two different time windows (Response selection and Amount spent), and thus two psychologically distinct decisional stages, we found that AIns was involved in deciding whether or not to react to injustice, whereas amygdala was involved in deciding how much to spend in order to react to injustice, both in SP and in TP. MPFC was also involved in determining the severity of the reaction, possibly reflecting the value computation, and the related affective valence, of the choice^30^. These results support the idea that the willingness to react to an injustice is driven by prosocial motivations such as a preference for equality^17,22,26^, whilst the severity of the reaction, whether it be punishing the offender or compensating the victim, reflects the affective component of the response to social norm violations^27,37,38^.

In summary, participants here demonstrated an equal preference for both punishment and compensation. ToM and perspective taking processes are more engaged in compensation, but neither option seems to be intrinsically rewarding per se; what is valuable is having the chance to react in the preferred way. Furthermore, we found support for two distinct psychological processes involved in the decision: unfairness perception, required to respond to an injustice, and the affective response to unfairness, associated with the severity of the reaction. Overall, the current study contributes to the developing body of research that aims to investigate the neuro-cognitive mechanisms underlying social behaviour, and, in particular, social norm compliance, a foundational aspect of a well-functioning society.

## Methods

### Participants

Forty right-handed participants (N(Males) = 7; M_age_ = 23,4 years; age range: 19–27) were recruited; two participants dropped out before undergoing (f)MRI, hence 38 completed the full task. Participants were paid €10 per hour, plus a potential small bonus depending on their choices. Exclusion criteria involved history of mental health problems and non-MRI compatible characteristics. The study was approved by the local regional ethics board (Commissie Mensgebonden Onderzoek Regio Arnhem-Nijmegen, CMO 2014/288 “Imaging Human Cognition”), in accordance with the Declaration of Helsinki regarding human experimentation. All participants provided written informed consent.

### Task

**Figure 5:**
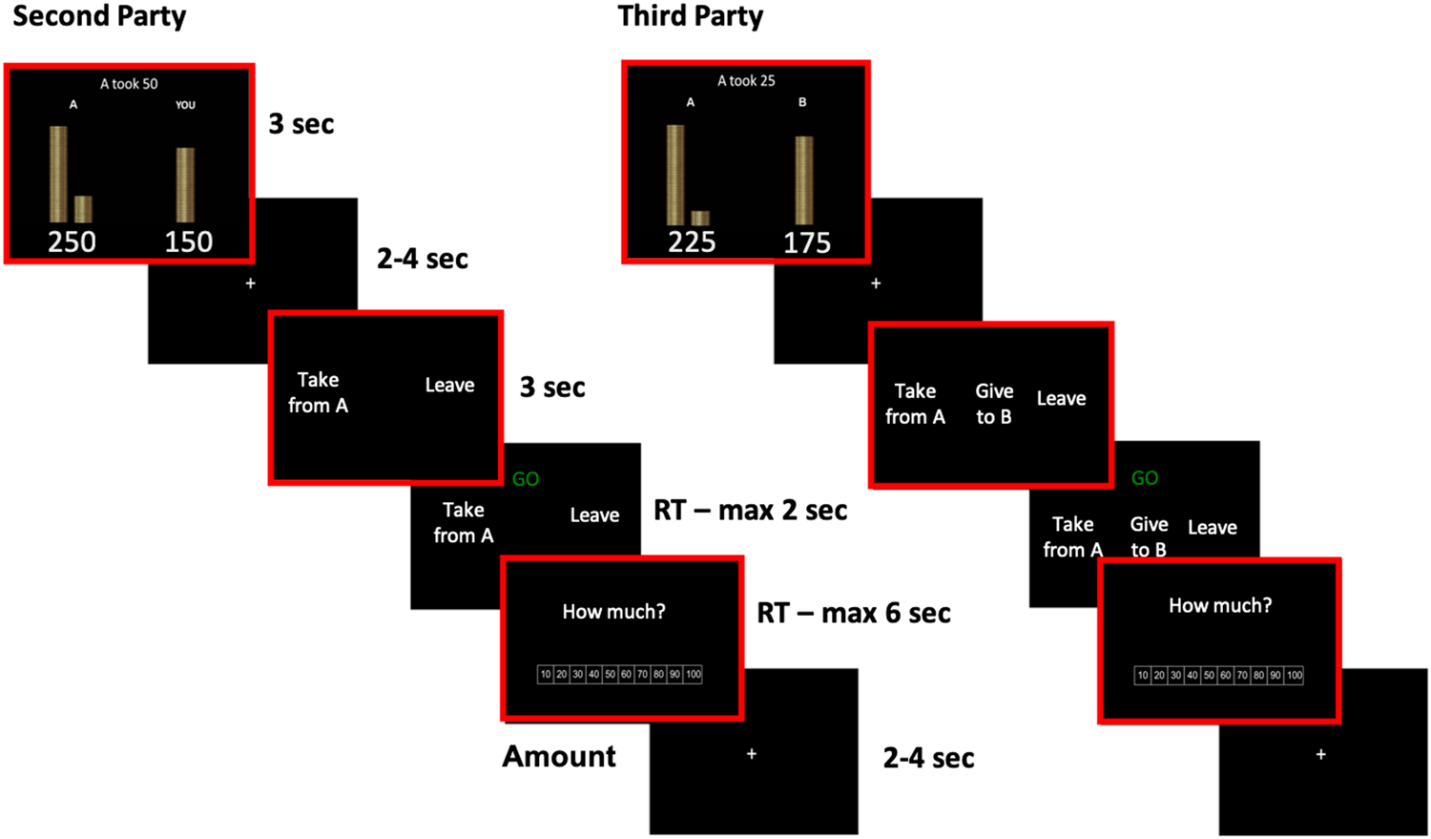
Justice Game task. Example of two Justice Game trials, in Second-Party and Third-Party conditions, where Player A took chips from the other player, and the participant decided to react to injustice. In the event of a “Leave” choice, the Amount screen would have been replaced by a screen asking to wait 3 seconds for the next trial. The time-windows of interest for the fMRI analysis are framed in red (*Division*, *Response* and *Amount*). The duration of the first two windows is fixed (3 seconds), while the *Go* and the *Amount* screen are self-paced (maximum duration of 2 and 6 seconds, respectively). The inter-stimulus interval between the *Division* and the *Response* screen and the inter-trial interval are jittered between 2 and 4 seconds.

A modified version of the Justice Game, presented to participants as “Chip Division Game” to avoid potential biases, was developed using Matlab Psychtoolbox^39–41^. All players started with 200 monetary units (Mus; 1MU = 1 eurocent). In one condition (Second Party, SP), participants faced an opponent (Player A) that could steal MUs from them; in a second condition (Third Party, TP), participants observed two players, one of whom (player A) could steal MUs from the other (player B). Half of the trials were fair (when player A took 0, with both A and B possessing 200) and half were unfair (when player A took MUs from the participant/player B, so player A had more MUs than the other player), with a varying level of injustice (25, 50, 75 or 100 MUs taken from player B). At this point, participants could decide to either do nothing (“Leave”) or spend some of their endowment (200 MUs) to change the payoffs. In SP, 40 trials were administered, and the choice was always between “Leave” or “Take from A”, which meant to reduce player A’s payoff. In TP, 120 trials were administered: 40 trials presented all three options, i.e., “Leave”, “Take from A”, “Give to B” (LTG trials), and 80 trials presented only two, either “Leave” and “Take from A” (40 LT trials), or “Leave” and “Give to B” (40 LG trials). As in SP, “Take from A” meant reducing Player A’s MUs, whereas “Give to B” meant increasing player B’s MUs. The options were presented for 3 seconds before a green sign “Go!” appeared on the screen, prompting participants to enter their decision via a response box. If the choice was “Take from A” or “Give to B”, participants were required to indicate how much they wanted to spend to punish or compensate. Consistent with previous studies^6,8,11^, the punishment (compensation) ratio was 1:3, that is, if participants spent 10 MUs to punish (compensate) player A (B), player A (B) would lose (gain) 30 MUs. Figure 5 represents an example of an unfair trial. SP and TP conditions were presented to all participants in two separate blocks, with a counterbalanced order. Participants were told players A and B were real and had played in previous sessions, although all trials were pre-programmed. Participants were also told to treat each trial independent from the others, as each one represented a different pair of players (multiple one-shot trials). At the end, two trials were randomly selected to determine participants’ final payoff, as per the conversion rate described above.

### Procedure

Upon arrival at the Donders Centre for Cognitive Neuroimaging’s fMRI facilities, participants were given both the informed consent and the MRI-compatibility checklist to fill out and sign. Then, they were taken in an experimental room for instructions and practice trials, before being placed into the MRI scanner. Depending on the presentation order of the conditions, participants were trained on either SP or TP condition outside the scanner, where they read the instructions and then answered some questions to ensure the understanding of the rules. For example, for the SP condition, participants were asked: “Player A took 50 chips from you. If you chose to spend 50 chips to take from player A, what will the final outcome (in chips) for A and yourself be?”. Participants would answer and be given the chance to discuss with the experimenter any doubts or uncertainties that may have arisen. After that, they would do 5 training trials, to become familiar with the response mode. The condition they were trained on outside the scanner was the condition with which the fMRI session would begin. The second condition was only explained inside the scanner immediately before playing it, and once the other condition was completed, to decrease demand characteristics and the risk of potentially biasing participants’ choices. A total of 160 trials was administered, for a maximum task time length of 55 minutes. A brief break was provided after 20 minutes. A structural image was acquired at the end. Overall, participants spent approximately one hour and 15 minutes inside the MRI scanner.

### fMRI analysis

#### fMRI data acquisition and pre-processing

A 3T Siemens Magnetom Prisma MRI scanner collected functional and structural brain images. Functional images were collected using a multi echo T2*-weighted sequence. Thirty-five functional images per volume were collected in an ascending, interleaved manner (flip angle 90°, TR 2250 ms, TE 9.4 ms, 8.9 ms 8.4 ms and 7.9 ms, field of view: 224 mm, voxel size 3 × 3 × 3 mm and 0.5 mm slice gap), covering the entire cortex. High-resolution T1-weighted structural images (MPRAGE, 192 slices, TR 2300 ms, TE 3.03 ms, voxel size 1 × 1 × 1 mm) were also acquired.

Images were processed and analysed with SPM 12^42^. Images were time corrected and realigned to the first volume in order to correct for motion artefacts. The T1 image was co-registered to the mean functional image and segmented into tissue types. Parameters from segmentation were used to normalize all images to the MNI 152 brains template (12-parameter affine transformation); images were then spatially normalized into a standard space (MNI template), resliced to 3 mm isotropic voxels and smoothed with a 6 mm FWHM Gaussian kernel.

#### fMRI GLMs specifications

Two general linear models were specified at the first level, including run (one SP block, two TP blocks) as a fixed effect, 6 realignment parameters as regressors of no interests, and time derivatives for each parameter. Parameters were convolved with a double-gamma canonical hemodynamic response function (HRF). All contrasts of interests were estimated at the first-level (subject level), and then fed into a second-level analysis using a mixed-effect general linear model.

The *Options* model focused on the effects of the availability of different options to react to injustice, as well as on the correlates of unfairness perception. Blood Oxygenated Level Dependent (BOLD) signal was estimated for each participant, for each run and for each voxel as follows:

$$\begin{array}{c}{\rm{BOLD}}={\beta }_{0}+{\beta }_{1}DivS{P}_{fair}+{\beta }_{2}DivT{P}_{fair}+{\beta }_{3}DivS{P}_{unfair}\\ \,\,\,\,\,++\,{\beta }_{4}DivS{P}_{unfair}\ast Injustic{e}_{param}+{\beta }_{5}DivT{P}_{unfair}\\ \,\,\,\,\,+{\beta }_{6}DivT{P}_{unfair}\ast Injustic{e}_{param}+{\beta }_{7}RespSP\\ \,\,\,\,\,+{\beta }_{8}RespTP\_LT+{\beta }_{9}RespTP\_LG\\ \,\,\,\,\,+{\beta }_{10}RespTP\_LTG+{\beta }_{11}Go+{\beta }_{12}Wait/Am\end{array}$$

where *Div*_*fair*_ and *Div*_*unfair*_ (*SP* or *TP*) indicate a fair or unfair division screen; *injustice*_*param*_ a parametric modulator of injustice (chips Player A took from the other player); *Resp* a response screen, with *TP_LT* and *TP_LG* referring to trials in which participants only had two options, i.e., leave or take from A (*TP_LT*), and leave or give to B (*TP_LG*), and *TP_LTG* referring to trials in which participants had all the three options available; *Go* the Go screen, and *Wait/Am* the screen following a decision (i.e., wait for the next trial after a Leave decision, or select an amount after a Take or Give decision). All 38 participants were included in this analysis.

The contrasts of interests estimated for the *Options* model were: *Div*_*Unfair*_*-Div*_*Fair*_ (main effect of Injustice, grouping SP and TP) to determine unfairness-related areas; *DivSP-DivTP* (main effect of Target, grouping unfair and fair divisions) to determine target-related areas; *DivSP*_(__*Unfair-Fair*)_*-DivTP*_(__*Unfair-fair*)_ (interaction) to determine whether unfairness-related areas were more involved in SP compared to TP; *RespSP-RespTP_LT*, to determine areas that are more involved in SP responses compared to TP – note that for TP, the analysis was restricted to LT trials (only-punishment option) in order to keep it comparable to SP; *RespTP_LT-RespTP_LG*, and the reverse, to determine areas that are more involved in deciding whether to punish or leave compared to deciding whether to compensate or leave.

In order to compare individual preferences for punishment and compensation, participants were divided into Compensators and Punishers; those who chose compensation more often than punishment in TP_LTG trials (i.e., when all options were available), would be classified as Compensators (N = 19); vice versa, those who preferred punishment to compensation would be classified as Punishers (N = 14).

A full-factorial second-level design was run on the *Response* time-window, considering Preference (Compensators and Punishers) as a between-subject factor and Option (TP_LTG, TP_LT, TP_LG) as a within-subject factor. Contrasts of interests considered were (*Punishers – Compensators*) and reverse, (*Compensators_LG* + *Punishers_LT*) − *rest*, and (*Compensators_LT* + *Punishers_LG*) − *rest*. These contrasts tested the distinct neural processes involved when participants were given the opportunity to choose their preferred option, and when they could only choose their non-preferred option in order to react to injustice.

The *Choice* model focused on participants’ choices, i.e., punish or compensate, and the amount spent to do so. BOLD signal was estimated for each participant, for each run and for each voxel as follows:

$$\begin{array}{rcl}{\rm{BOLD}} & = & {\beta }_{0}+{\beta }_{1}DivSP\_fair+{\beta }_{2}DivSP\_unfair\\  &  & +{\beta }_{3}DivTP+{\beta }_{4}DivT{P}_{fair}\\  &  & +{\beta }_{5}DivT{P}_{unfair}+{\beta }_{6}RespS{P}_{leave}\\  &  & +{\beta }_{7}RespT{P}_{leave}+{\beta }_{8}RespS{P}_{pun}\\  &  & +{\beta }_{9}RespT{P}_{pun}+{\beta }_{10}RespT{P}_{comp}\\  &  & +{\beta }_{11}Go+{\beta }_{12}Wait+{\beta }_{13}AmS{P}_{pun}\\  &  & +{\beta }_{14}AmS{P}_{pun}\ast S{P}_{pu{n}_{param}}+{\beta }_{15}AmT{P}_{pun}\\  &  & +{\beta }_{16}AmT{P}_{pun}\ast T{P}_{pu{n}_{param}}+{\beta }_{17}AmT{P}_{comp}\\  &  & +{\beta }_{18}AmT{P}_{comp}\ast T{P}_{com{p}_{param}}\end{array}$$

Where *RespSP*_*leave*_ and *RespSP*_*pun*_ indicate the choice to leave and to punish in SP; *RespTP*_*leave*_, *RespTP*_*pun*_ and *RespTP*_*comp*_ the decisions in TP; *Wait* the screen after a leave decision; *AmSP*, *AmTP*_*pun*_ and *AmTP*_*comp*_ the amount screen; *SPpun*_*param*_, *TPpun*_*param*_ and *TPcomp*_*param*_ the parametric modulators indicating the amount spent for SP punishment, TP punishment and TP compensation respectively. Only participants who had at least 3 trials for each type of response were included in this analysis^8^, and thus the sample was reduced to N = 20.

The contrasts estimated for the *Choice* model were: *RespTP*_*pun*_*-RespTP*_*comp*_, and the reverse, to determine the simple effect of punishment vs compensation and viceversa; *RespSP*_*pun*_*-RespTP*_*pun*_, and the reverse, to determine the areas involved in SP vs TP punishment, and viceversa. The contrasts [*Resp_TP*(*pun* + *comp*) + *Resp_SP*] − [*AmTP*(*pun* + *comp*)_*param*_ + *AmSP*_*param*_], and reverse, comparing the *Response* screen and the *Amount* screen, parametrically modulated by the actual amount spent, were run in order to analyse the areas involved in response selection and areas involved in the severity of the reaction. TP and SP were collapsed because the focus here was on the reaction itself rather than the target.

For both models, all parameters were modelled as 3-sec boxcars, except the *Go* and the *Wait* and *Amount* screens, which were modelled as stick functions.

The datasets generated are available from the corresponding author on any reasonable request.
